# Incidence and types of congenital heart disease at a referral hospital in Jordan: retrospective study from a tertiary center

**DOI:** 10.3389/fped.2023.1261130

**Published:** 2023-09-15

**Authors:** Abeer A. Hasan, Naser Aldain A. Abu Lehyah, Moath K. Al Tarawneh, Mahmoud Y. Abbad, Areen G. Fraijat, Razan A. Al-Jammal, Dania M. Moamar, Qasem A. Shersheer, Scott O. Guthrie, Joseph R. Starnes

**Affiliations:** ^1^ Division of Neonatology, Department of Pediatrics, Maternity and Children’s Hospital at Al Bashir Hospital, Amman, Jordan; ^2^ Department of General Pediatrics, Maternity and Children’s Hospital at Al Bashir Hospital, Amman, Jordan; ^3^ Division of Pediatric Critical Care Medicine, Department of Pediatrics, Maternity and Children’s Hospital at Al Bashir Hospital, Amman, Jordan; ^4^ Department of Obstetrics and Gynaecology, Maternity and Children’s Hospital at Al Bashir Hospital, Amman, Jordan; ^5^ Division of Neonatology, Department of Pediatrics, Vanderbilt University Medical Center, Nashville, TN, United States; ^6^ Division of Pediatric Cardiology, Department of Pediatrics, Vanderbilt University Medical Center, Nashville, TN, United States

**Keywords:** Jordan, congenital heart defects, cardiovascular disease, epidemiology, septal defects

## Abstract

**Background:**

Congenital heart disease (CHD) is the most common birth defect and accounts for significant global morbidity and mortality. Relatively little is known about the epidemiology of CHD in Jordan or the manner in which CHD is identified.

**Methods:**

A retrospective medical record review was conducted for all neonates who had an abnormal echocardiogram performed at a tertiary referral hospital. All included neonates had echocardiography performed by the same pediatric cardiologist at the discretion of the treatment team. Descriptive statistics were used to describe CHD incidence, types of CHD identified, and mechanism of identification.

**Results:**

The incidence of congenital heart disease was 17.8 per 1,000 live births. This rose to 24.6 per 1,000 if patent ductus arteriosus in preterm infants was included. The most common identified abnormalities were PDA, atrial septal defects, persistent pulmonary hypertension, septal hypertrophy, and ventricular septal defects. Most children were evaluated either for a murmur heard on exam or as a part of screening due to other comorbidities or risk factors. Less than 1% of children had a prenatal diagnosis. There was a higher rate of persistent pulmonary hypertension during the COVID-19 pandemic than before (*p* < 0.001).

**Conclusions:**

There is a high incidence of CHD in Jordan. Increased prenatal and perinatal screening for CHD may allow for earlier detection.

## 1. Introduction

Congenital heart disease (CHD) is the most common birth defect, and it is often stated that the incidence of CHD globally is 8 per 1,000 live births (1, 2) with more recent studies finding increases to as high as 9.5 per 1,000 (3). This overall estimate masks significant regional variability. Although varying definitions make quantification difficult, regional studies have found incidences ranging from 1.2 to 17 per 1,000 live births (2). Reported incidences have also increased over time as detection has improved, and it is now estimated that 1.35 million children are born annually with CHD (1). CHD accounted for at least 260,000 deaths in 2017, including 180,000 deaths among infants (4). There is also marked regional inequality in mortality, with mortality rates from CHD correlating closely with country-level socioeconomic indicators. Improving mortality from CHD will be integral to achieving the Sustainable Development Goals (SDGs), including SDG 3.2 to reduce neonatal mortality and SDG 3.4 to reduce premature deaths from non-communicable diseases (5).

Ultimate diagnosis of CHD comes via cardiac imaging, usually echocardiogram. Prenatal diagnosis via fetal echocardiogram is increasingly common in high-resource settings. Detection rates vary greatly—from less than 10% to greater than 80%—depending on setting and cardiac lesion with the lowest detection rates for pulmonary venous anomalies (6). In middle-income countries, access to fetal echocardiography is rare. Even among centers performing congenital heart surgeries, less than half report always having fetal echocardiography available when needed (7).

Screening for critical CHD via pulse oximetry has recently been utilized to capture patients earlier when disease is not prenatally detected. Meta-analyses have found specificity for critical CHD of 99.9%, sensitivity of about 76%, and a false positive rate of 0.14% (8, 9). While many high-income countries now have mandatory screening, challenges in implementation in low- and middle-income countries (LMICs) have been encountered (10, 11). This means many children are identified later by astute clinicians or when symptoms develop. This leads to important delays in treatment that may lead to worse outcomes (12).

Relatively little is known about the epidemiology of CHD in Jordan. There have been four single-center studies to date (13–16). Two studies have reported incidence of CHD with rates ranging from 12.3 per 1,000 live births (13) to 25 per 1,000 (14). Both are substantially higher than internationally reported incidences. Three studies found that ventricular septal defects (VSDs) were the most common abnormality, accounting for more than 40% of CHD (14–16). A fourth study found patent ductus arteriosus (PDA) to be the most common (13). Most children ultimately diagnosed with CHD were referred for evaluation based on murmur or cyanosis (13). CHD is an important factor in both neonatal and childhood mortality in Jordan (17, 18). Fetal echocardiography is available at some referral centers (19). Our goal is to report on the incidence of CHD noted at the largest birthing hospital in Jordan using the largest cohort reported to date in the country.

## 2. Materials and methods

### 2.1. Study setting

The Hashemite Kingdom of Jordan is a middle-income country located in the Levant. Al Bashir Hospital is the largest medical center in the country and is located in the capital city of Amman. Al Bashir is composed of five distinct hospitals, houses multiple residency programs, and serves as a teaching hospital for three major universities. The Maternity and Children's Hospital at Al Bashir Hospital is a tertiary referral hospital with a capacity of 450 beds, including a neonatal intensive care unit (NICU) with approximately 100 beds. It receives referrals from most hospitals throughout the country. There were no fetal maternal medicine doctors present at Al Bashir Hospital during the study period. Of note, the data were partially collected during the coronavirus disease 2019 (COVID-19) pandemic. Many women did not receive prenatal care until late in pregnancy due to local and national COVID-19 quarantine and isolation policies.

In Al Bashir hospital, the pediatric cardiology team is composed of a senior pediatric cardiology consultant and a pediatric cardiology specialist, who together provide detailed cardiac assessment and echocardiogram evaluation to more than 30 patients daily and more then 5,000 patients per year. These evaluations are primarily performed as a part of outpatient clinics. Pediatric cardiology also participates in the evaluation of neonatal and pediatric intensive care unit patients daily. These inpatients are seen after initial evaluation by the primary neonatologist or general pediatric specialist. Pulse oximetry screening for CHD has been mandatory for all neonates in the Al Bashir NICU since 2020. Inpatient evaluations often include chest x-ray, hyperoxia tests, and echocardiograms.

### 2.2. Data collection

A retrospective medical record review was conducted for all neonates who had an abnormal echocardiogram performed in the NICU of Al Bashir Hospital during the three-year period beginning in January 2019 and ending in December 2021. All included neonates had two-dimensional echocardiography with Doppler performed by the same pediatric cardiologist. Echocardiograms were obtained at the discretion of clinical providers during the course of regular care.

Data were collected using a standardized data collection tool. The total number of deliveries, NICU admissions, and echocardiograms performed were tracked. Further chart review was conducted for children with abnormal echocardiograms. For these children, variables abstracted from the chart included year of birth, infant sex, gestational age, birth weight, the presence of other congenital anomalies, and disposition. The indication for echocardiogram and ultimate diagnosis were also collected. Cases of isolated patent foramen ovale (PFO) were not considered abnormal as this is considered a normal anatomic variant.

### 2.3. Statistical analysis

Descriptive statistics are presented as median with interquartile range (IQR) for continuous variables and percentages for categorical variables. Rates were calculated as simple fractions. All analyses were performed using Stata version 14.2 (StataCorp LP, College Station, TX, USA). For the calculation of congenital heart disease incidence, the definition used by the Global Burden of Disease study was utilized (4). This excluded patients with persistent pulmonary hypertension, myocardial hypertrophy, arrhythmia, and intracardiac masses. Similarly, PDA in term infants was not included as this can be physiologic during the first several days of life. Additionally, a small number of infants (8, 0.5%) included in the primary analysis were excluded from rate calculations because they presented in shock after discharge from other hospitals.

## 3. Results

### 3.1. Demographics

There were 43,420 deliveries during the three-year study, and 3,317 echocardiograms were performed. There were 1,497 abnormalities noted on echocardiogram for an abnormal rate of 34.5 per 1,000 live births. There was a slight male predominance at 55% (Table 1). The majority of babies were pre-term overall. When limited to babies who did not have patent ductus arteriosus, the majority of babies were term (60.4%). Most neonates had acyanotic heart disease, but a substantial minority (12.0%) had cyanotic disease. The incidence of congenital heart disease was 17.8 per 1,000 live births with patent ductus arteriosus excluded. If patent ductus arteriosus in preterm infants was included, this rose to 24.6 per 1,000 live births. There were 97 children (6.5%) who died prior to discharge. The most common cause of death was hypoplastic left heart syndrome, which was universally fatal among 25 infants.

**Table 1:** Demographics of patients with abnormal echocardiograms.

Year	
2019	460 (30.7%)
2020	468 (31.3%)
2021	569 (38.9%)
Sex	
Female	673 (45.0%)
Male	824 (55.0%)
Gestational age (weeks)	37 (34, 40)
Term	
Term (≥38 weeks)	728 (48.6%)
Pre-term	769 (51.4%)
Birthweight (kg)	2.7 (1.9, 3.2)
Type of cardiac disease	
Acyanotic	899 (60.1%)
Cyanotic	179 (12.0%)
Other	419 (28.0%)
Disposition	
Discharged	1,351 (90.2%)
Deceased	97 (6.5%)
Transferred to heart center	49 (3.3%)

Continuous as median (IQR); categorical as *N* (%).

### 3.2. Identified cardiac disease

The most common identified abnormalities were patent ductus arteriosus (PDA), atrial septal defects (ASD), persistent pulmonary hypertension (PPH), septal hypertrophy, and ventricular septal defects (VSD) (Table 2). The most common cyanotic heart disease was Tetralogy of Fallot.

**Table 2:** Cardiac abnormalities identified.

	Total (*N* = 1,497)	CHD (*N* = 1,078)
**Acyanotic**	**899** (**60.1%)**	**899** (**83.4%)**
AV septal defect: complete	38 (2.5%)	38 (3.5%)
AV septal defect: intermediate	23 (1.5%)	23 (2.1%)
AV septal defect: transitional	11 (0.7%)	11 (1.0%)
Aortic stenosis	6 (0.4%)	6 (0.6%)
Atrial septal defect	236 (15.8%)	236 (21.9%)
Coarctation of aorta	22 (1.5%)	22 (2.0%)
Dextrocardia	11 (0.7%)	11 (1.0%)
Patent ductus arteriosus (preterm)	295 (19.7%)	295 (27.4)
Pulmonary stenosis	74 (4.9%)	74 (6.9%)
Ventricular septal defect	183 (12.2%)	183 (17.0%)
**Cyanotic**	**179** (**12.0%)**	**179** (**16.6%)**
Critical pulmonary stenosis	2 (0.1%)	2 (0.2%)
Double outlet right ventricle	15 (1%)	15 (1.4%)
Hypoplastic left heart syndrome	25 (1.7%)	25 (2.3%)
Pulmonary atresia	9 (0.6%)	9 (0.8%)
Single ventricle	20 (1.3%)	20 (1.9%)
Tetralogy of Fallot	42 (2.8%)	42 (3.9%)
Total anomalous pulmonary venous return	18 (1.2%)	18 (1.7%)
Transposition of great vessels	28 (1.9%)	28 (2.6%)
Tricuspid atresia	6 (0.4%)	6 (0.6%)
Truncus arteriosus	14 (0.9%)	14 (1.3%)
**Other**	**419** (**28.0%)**	–
Hypertrophic cardiomyopathy	5 (0.3%)	–
Persistent pulmonary hypertension	189 (12.6%)	–
Patent ductus arteriosus (term)	21 (1.4%)	–
Ruptured sinus of Valsalva	1 (0.1%)	–
Second degree heart block	7 (0.5%)	–
Septal hypertrophy	186 (12.4%)	–
Supraventricular tachycardia	9 (0.6%)	–
Tricuspid valve mass	1 (0.1%)	–

Among preterm infants with PDA, all 295 received ibuprofen to attempt medical closure. A total of 163 (55.3%) received three doses, 126 (42.7%) received six doses, and six (2.0%) received nine doses. The majority (278, 94.2%) closed on subsequent imaging.

There were 34 cases of PPH identified among 16,309 births in 2019 giving a rate of 2.08 per 1,000 live births. In 2021, there were 107 cases among 14,327 births giving a rate of 7.47 per 1,000 live births. The rate was higher in 2021 than in 2019 (*p* < 0.001).

A total of 409 (27.3%) children had at least one major comorbidity. The most common comorbidity was maternal diabetes, which accounted for 253 (16.9%) children. Most children of diabetic mothers had septal hypertrophy (184, 72.7%). Down Syndrome was also common with 58 (3.9%) neonates diagnosed. The most common defects in this group were atrioventricular septal defects (24, 41.4%).

### 3.3. Indication for cardiac evaluation

Most children were evaluated either for a murmur heard on exam or as a part of screening (Table 3). Only 11 children, representing less than 1% of identified disease, were diagnosed prenatally.

**Table 3:** Indication for cardiology evaluation.

Indication	*N* (%)
Bradycardia	2 (0.1%)
Chest x-ray abnormality	18 (1.2%)
Echo screening^a^	411 (27.4%)
Echo screening for PDA	13 (0.9%)
Family history of CHD	1 (0.1%)
Heart murmur	571 (38.1%)
Prenatal diagnosis	11 (0.7%)
Respiratory distress	70 (4.7%)
Failed pulse oximetry screening	196 (13.1%)
Shock^b^	8 (0.5%)
Tachypnea or tachycardia	24 (1.6%)
Weak pulses	5 (0.3%)
Unknown	167 (11.2%)

^a^ Screening due to congenital abnormalities, infants of diabetic mothers, other syndromes.

^b^ Admitted after birth and not born at Al Bashir.

## 4. Discussion

This is the largest study to date of CHD incidence in Jordan, and we identified a high incidence of CHD at a major tertiary care center. The calculated incidence (24.6 per 1,000 live births with preterm PDA; 17.8 per 1,000 without PDA) is higher than internationally reported averages (1, 2). These high rates are similar to those previously reported in similar studies from Jordan (13, 14). This may reflect the study setting at a tertiary center but may also suggest a higher underlying rate of CHD compared to other settings. Regardless, this high incidence of CHD necessitates ongoing investment in the detection and management of CHD at Al Bashir Hospital and in Jordan more generally. This is further reinforced by high in-hospital death rates, especially among critical CHD such as hypoplastic left heart syndrome.

The reason for this high rate of CHD requires additional investigation. High rates of consanguinity have been identified as a risk factor for CHD in other regional countries (20, 21) and in systematic reviews (22). This has been a proposed etiology for the high rates of CHD (13) and other congenital anomalies (23) in Jordan. It is also possible that environmental, ethnic, or socioeconomic differences account for this increased rate of CHD (1). For example, there is some evidence of varying prevalence of specific lesions across ethnic groups (1, 24). There are also a wide variety of environmental exposures that have been linked with CHD (25). Some authors have observed an increase in PPH during the COVID-19 pandemic (26), which may have played a role in our high rates of PPH as well given that we observed higher rates of PPH in 2021 during the pandemic than in 2019 before it began.

The most commonly identified abnormalities were premature PDA (27.4%), ASD (21.9%), and VSD (17.0%). While PDA was the most commonly identified lesion in one other population-based study from Jordan (13), PDA represented less than 12% of lesions in the three other available studies (14–16). All three of these studies found VSD to be the most common, representing more than 40% of CHD identified. While the high rate of PDA in our study is likely, at least in part, related to the NICU setting and high prevalence of prematurity, this would not account for the relatively high rate of ASD and lower rate of VSD in our population. This likely represents true variability in the populations served by the hospitals conducting these studies. Importantly, the majority of PDAs identified were able to be medically closed with ibuprofen.

Nearly 40% of infants were referred for cardiology evaluation based on a murmur heard on exam, and nearly another 30% were referred based on other congenital abnormalities or maternal risk factors. Less than 1% had a prenatal diagnosis of CHD. This is much lower than the prenatal detection rate when widespread screening is available, which can be near 60% (27, 28). This is likely due to lack of availability of prenatal echocardiography at Al Bashir Hospital. Infants with prenatal diagnoses were either diagnosed in a private institution or on routine obstetric anatomy scans performed by general obstetricians. This is similar to other middle-income countries, where prenatal echocardiography is difficult to access. Among hospitals performing congenital heart surgeries in middle-income countries, fetal echocardiography is reportedly only available and performed about half the time when CHD is suspected (7). Because reduced morbidity and mortality have been described with prenatal diagnosis across various heart lesions (29), this represents a significant potential area for improvement both in Jordan and LMICs more generally. Importantly, some congenital heart disease described in this study cannot be detected prenatally, for example, PDA and ostium secundum ASDs, which are physiologic *in utero*.

More than 10% of children with abnormal echocardiograms were referred because of failed pulse oximetry screening. This included 125 children with cyanotic heart disease that may not otherwise have been identified before discharge. Pulse oximetry screening was introduced at Al Bashir Hospital during the study period in 2020. Failure to diagnose critical CHD prior to discharge can lead to increased morbidity and mortality (30), and widespread implementation of pulse oximetry screening has been associated with a decrease in mortality (31). Further implementation of screening with pulse oximetry in Jordan may lead to reductions in mortality.

### 4.1. Limitations

Our study is limited by the setting in a referral NICU. This makes quantification of the community incidence of CHD difficult, as infants who are ill are referred to the institution. This has likely increased our estimate of incidence. We also do not have data regarding maternal COVID-19 infection, which limits our ability to comment on the association between maternal COVID-19 and PPH. Additionally, data regarding the size of septal defects were not available, limiting our ability to describe the severity of these lesions. Finally, data are not available about the results of pulse oximetry screening for children who were not referred for cardiology evaluation. This makes quantification of the sensitivity, specificity, and false positive values for pulse oximetry testing in this setting impossible. Future research will prospectively collect these data to address this limitation and is currently underway.

## 5. Conclusion

This study identifies a high incidence of CHD at a major tertiary care center in Jordan and describes the most common identified disease. Most children were referred for evaluation based on clinical presentation or examination, and very few were referred based on prenatal diagnosis or pulse oximetry screening. Increased prenatal and perinatal screening for CHD may allow for earlier detection.

## Data Availability

The raw data supporting the conclusions of this article will be made available by the authors, without undue reservation.

## References

[1] van der Linde D Konings EEM Slager MA Witsenburg M Helbing WA Takkenberg JJM Birth prevalence of congenital heart disease worldwide: a systematic review and meta-analysis. J Am Coll Cardiol. (2011) 58:2241–7. 10.1016/j.jacc.2011.08.025 22078432

[2] Bernier P-L Stefanescu A Samoukovic G Tchervenkov CI. The challenge of congenital heart disease worldwide: epidemiologic and demographic facts. Semin Thorac Cardiovasc Surg Pediatr Card Surg Annu. (2010) 13:26–34. 10.1053/j.pcsu.2010.02.005 20307858

[3] Liu Y Chen S Zühlke L Black GC Choy M-K Li N Global birth prevalence of congenital heart defects 1970–2017: updated systematic review and meta-analysis of 260 studies. Int J Epidemiol. (2019) 48:455–63. 10.1093/ije/dyz009 30783674 PMC6469300

[4] Zimmerman MS Smith AGC Sable CA Echko MM Wilner LB Olsen HE Global, regional, and national burden of congenital heart disease, 1990–2017: a systematic analysis for the global burden of disease study 2017. Lancet Child Adolesc Health. (2020) 4:185–200. 10.1016/S2352-4642(19)30402-X 31978374 PMC7645774

[5] The United Nations. Sustainable development goals. Available at: http://www.un.org/sustainabledevelopment/sustainable-development-goals/ (Accessed June 30, 2017).

[6] Sun HY. Prenatal diagnosis of congenital heart defects: echocardiography. Transl Pediatr. (2021) 10:2210–24. 10.21037/tp-20-164 34584892 PMC8429868

[7] Majeed A Jenkins K Gauvreau K Forero JF Pérez Juárez F Kulkarni S Screening and diagnostic imaging at centres performing congenital heart surgery in middle-income countries. Cardiol Young. (2023) 33:780–6. 10.1017/S1047951122001731 35684953

[8] Plana MN Zamora J Suresh G Fernandez-Pineda L Thangaratinam S Ewer AK. Pulse oximetry screening for critical congenital heart defects. Cochrane Database Syst Rev. (2018) 3(3):CD011912. 10.1002/14651858.CD011912.pub2 29494750 PMC6494396

[9] Thangaratinam S Brown K Zamora J Khan KS Ewer AK. Pulse oximetry screening for critical congenital heart defects in asymptomatic newborn babies: a systematic review and meta-analysis. Lancet. (2012) 379:2459–64. 10.1016/S0140-6736(12)60107-X 22554860

[10] Abbas A Ewer AK. New born pulse oximetry screening: a global perspective. Early Hum Dev. (2021) 162:105457. 10.1016/j.earlhumdev.2021.105457 34548207

[11] Martin GR Ewer AK Gaviglio A Hom LA Saarinen A Sontag M Updated strategies for pulse oximetry screening for critical congenital heart disease. Pediatrics. (2020) 146:e20191650. 10.1542/peds.2019-1650 32499387

[12] Brown KL. Delayed diagnosis of congenital heart disease worsens preoperative condition and outcome of surgery in neonates. Heart. (2006) 92:1298–302. 10.1136/hrt.2005.078097 16449514 PMC1861169

[13] Khasawneh W Hakim F Abu Ras O Hejazi Y Abu-Aqoulah A. Incidence and patterns of congenital heart disease among Jordanian infants, a cohort study from a university tertiary center. Front Pediatr. (2020) 8:219. 10.3389/fped.2020.00219 32432065 PMC7214919

[14] Al-Ammouri I Ayoub F Tutunji L. Incidence of congenital heart disease in Jordanian children born at Jordan university hospital; a seven-year retrospective study. Jordan Med J. (2017) 51:109–17.

[15] Oweis N. Patterns of Congenital Heart Disease in Northern Jordan. Jordan Med J. (2006) 40:262–5.

[16] Amro K. Pattern of congenital heart disease in Jordan. Eur J Gen Med. (2009) 6:161–5.

[17] Daher AH Al-Ammouri I Ghanem N Abu Zahra M Al-Zayadneh E Al-Iede M. All-cause mortality in a pediatric intensive care unit at a teaching hospital in Amman, Jordan. Pediatr Int Off J Jpn Pediatr Soc. (2022) 64:e14940. 10.1111/ped.14940 34331816

[18] Abu-Heija AT. Causes and factors affecting perinatal mortality at Princess Basma Teaching Hospital in north Jordan. Asia Oceania J Obstet Gynaecol. (1994) 20:415–8. 10.1111/j.1447-0756.1994.tb00490.x 7832675

[19] Tutunji L Thekrallah F Basha A Awaysheh B Amer S Khatib L Prenatal detection of fetal heart disease at Jordan University Hospital: early experience in a developing country. Cardiol Young. (2019) 29:1072–7. 10.1017/S1047951119001550 31287035

[20] Becker SM Al Halees Z Molina C Paterson RM. Consanguinity and congenital heart disease in Saudi Arabia. Am J Med Genet. (2001) 99:8–13. 10.1002/1096-8628(20010215)99:1<8::aid-ajmg1116>3.0.co;2-u 11170087

[21] Al-Fahham MM Ali YA. Pattern of congenital heart disease among Egyptian children: a 3-year retrospective study. Egypt Heart J. (2021) 73:11. 10.1186/s43044-021-00133-0 33512632 PMC7846646

[22] Shieh JTC Bittles AH Hudgins L. Consanguinity and the risk of congenital heart disease. Am J Med Genet A. (2012) 158A:1236–41. 10.1002/ajmg.a.35272 22488956 PMC3331952

[23] Aqrabawi HE. Facial cleft and associated anomalies: incidence among infants at a Jordanian medical centre. East Mediterr Health J. (2008) 14:356–9. 18561727

[24] Jacobs EGJ Leung MP Karlberg J. Distribution of symptomatic congenital heart disease in Hong Kong. Pediatr Cardiol. (2000) 21:148–57. 10.1007/s002469910025 10754087

[25] Boyd R McMullen H Beqaj H Kalfa D. Environmental exposures and congenital heart disease. Pediatrics. (2022) 149:e2021052151. 10.1542/peds.2021-052151 34972224

[26] Ishqeir A Nir A Aptowitzer I Godfrey M; Pediatric Cardiology Unit, Shaare Zedek Medical Center, Jerusalem, Israel. Increased incidence of persistent pulmonary hypertension of the newborn following third trimester maternal COVID-19 infection. Eur Heart J. (2021) 42:ehab724.1843. 10.1093/eurheartj/ehab724.1843

[27] van Velzen C Clur S Rijlaarsdam M Bax C Pajkrt E Heymans M Prenatal detection of congenital heart disease-results of a national screening programme. BJOG. (2016) 123:400–7. 10.1111/1471-0528.13274 25625301

[28] Waern M Mellander M Berg A Carlsson Y. Prenatal detection of congenital heart disease—results of a Swedish screening program 2013–2017. BMC Pregnancy Childbirth. (2021) 21:579. 10.1186/s12884-021-04028-5 34420525 PMC8380393

[29] Simpson J. Impact of fetal echocardiography. Ann Pediatr Cardiol. (2009) 2:41. 10.4103/0974-2069.52806 20300268 PMC2840777

[30] Mahle WT Newburger JW Matherne GP Smith FC Hoke TR Koppel R Role of pulse oximetry in examining newborns for congenital heart disease: a scientific statement from the American Heart Association and American Academy of Pediatrics. Circulation. (2009) 120:447–58. 10.1161/CIRCULATIONAHA.109.192576 19581492

[31] Abouk R Grosse SD Ailes EC Oster ME. Association of US state implementation of newborn screening policies for critical congenital heart disease with early infant cardiac deaths. JAMA. (2017) 318:2111. 10.1001/jama.2017.17627 29209720 PMC5770276

